# Epac activation inhibits IL-6-induced cardiac myocyte dysfunction

**DOI:** 10.1007/s12576-016-0509-5

**Published:** 2016-12-19

**Authors:** Huiling Jin, Takayuki Fujita, Meihua Jin, Reiko Kurotani, Yuko Hidaka, Wenqian Cai, Kenji Suita, Rajesh Prajapati, Chen Liang, Yoshiki Ohnuki, Yasumasa Mototani, Masanari Umemura, Utako Yokoyama, Motohiko Sato, Satoshi Okumura, Yoshihiro Ishikawa

**Affiliations:** 1 Cardiovascular Research Institute, Yokohama City University Graduate School of Medicine, 3-9 Fukuura, Kanazawa-ku, Yokohama, 236-0004 Japan; 2 Department of Cardiac Physiology, National Cerebral and Cardiovascular Center Research Institute, 5-7-1 Fujishirodai, Suita-shi, Osaka, 565-8565 Japan; 3 Biochemical Engineering, Faculty of Engineering, Yamagata University, 4-3-16, Jonan, Yonezawa, Yamagata 992-8510 Japan; 4 Department of Physiology, Tsurumi University School of Dental Medicine, 2-1-3 Tsurumi, Tsurumi-ku, Yokohama, 230-8501 Japan; 5 Department of Physiology, Aichi Medical University, 1-1 Yazakokarimata, Nagakute, Aichi 480-1195 Japan

**Keywords:** Epac, cAMP, Catecholamine, Contractility, Cytokine, Jak-STAT

## Abstract

Pro-inflammatory cytokines are released in septic shock and impair cardiac function via the Jak-STAT pathway. It is well known that sympathetic and thus catecholamine signaling is activated thereafter to compensate for cardiac dysfunction. The mechanism of such compensation by catecholamine signaling has been traditionally understood to be cyclic AMP-dependent protein kinase (PKA)-mediated enforcement of cardiac contractility. We hypothesized that the exchange protein activated by cAMP (Epac), a newly identified target of cAMP signaling that functions independently of PKA, also plays a key role in this mechanism. In cultured cardiac myocytes, activation of Epac attenuated the inhibitory effect of interleukin-6 on the increase of intracellular Ca^2+^ concentration and contractility in response to isoproterenol, most likely through inhibition of the Jak-STAT pathway via SOCS3, with subsequent changes in inducible nitric oxide synthase expression. These findings suggest a new role of catecholamine signaling in compensating for cardiac dysfunction in heart failure. Epac and its downstream pathway may be a novel target for treating cardiac dysfunction in endotoxemia.

## Introduction

The classic cyclic AMP (cAMP)/protein kinase (PKA) pathway is a major regulator of cardiac function. Norepinephrine released from the synaptic terminals binds to β-adrenergic receptors, leading to activation of adenylyl cyclase and thus the production of cAMP [1, 2]. cAMP, a major second messenger, activates PKA, initiating cascades of phosphorylation reactions of molecules involved in myocyte contraction [3]. The final outcome of norepinephrine/cAMP signaling is to increase cardiac contractility. It is well known that sympathetic nerve activity, and thus cAMP production, is increased in response to decreased cardiac function in heart failure.

For many decades, it was believed that the major target of cAMP signaling is PKA and that the PKA-mediated increase in myocyte contractility is the only mechanism compensating for cardiac dysfunction in heart failure. Recently, however, exchange protein activated by cAMP (Epac) was identified as a new target of cAMP signaling that is activated independently of PKA [4, 5]. Epac has two isoforms (Epac1 and Epac2), and Epac1 is expressed dominantly in the heart [4]. Although the exact role of these molecules in regulating cardiac function is not fully understood, Epac has been shown to regulate various cellular functions, such as migration, proliferation, skeletal and masseter muscle hypertrophy, cardiac function, exocytosis or cell apoptosis, via regulation of Rap1, a small GTPase [4–11]. In cardiac myocytes, pharmacological activation of Epac induces myocyte hypertrophy [12, 13].

Increased production of cytokines, especially inflammatory cytokines such as tumor necrosis factor alpha (TNFα), interleukin-1 (IL-1) or IL-6, is responsible, at least in part, for cardiac dysfunction in patients with heart failure [14]. These inflammatory cytokines also play a role in septic shock, which is induced by exposure to bacterial endotoxin, leading to overproduction of these cytokines [15]. Cytokine-induced inducible nitric oxide synthase (iNOS) activation has been reported to play an important role in the development of septic cardiomyopathy [16, 17]. Septic cardiomyopathy is a well-described complication of severe sepsis, with more than two-thirds of patients exhibiting cardiac dysfunction after the onset of septic shock, even with a concomitant increase in sympathetic nerve activity [18, 19]. Interestingly, it is also known that the recovery of cardiac function is rapid and near-complete in septic shock, despite the severity of cardiac dysfunction, implying that activation of cAMP/Epac signaling may not merely increase cardiac contractility.

Activation of cytokine receptors is linked to the janus kinase–signal transducer and activator of transcription (Jak-STAT) pathway, which is a major intracellular signaling pathway [20]. Suppressor of cytokine signaling-3 (SOCS3) protein is induced upon cytokine receptor activation through STAT-dependent elements in the promoter. SOCS3 exerts negative feedback effects on cytokine signaling via the Jak-STAT pathway [21]. To date, interaction between the sympathetic nervous system and pro-inflammatory cytokine signaling remains poorly understood, despite the fact that both play an important role in the development of heart failure.

Recent studies have demonstrated that Epac regulates cytokine signaling in macrophages and endothelial cells [22–24]. We thus hypothesized that a novel cAMP/Epac signaling mechanism regulates cardiac function in heart failure, working independently of the PKA-mediated enforcement of cardiac contractility. In the study reported here, we show that activation of the cAMP/Epac pathway protects the cardiac myocytes against cytokine-induced dysfunction, most likely through inhibition of the Jak-STAT pathway via SOCS3, with subsequent changes in iNOS expression. These results suggest that Epac plays a key role in pro-inflammatory cytokine-induced cardiac dysfunction.

## Materials and methods

### Reagents

All chemicals were purchased from Sigma (St. Louis, MO), except for 8-(4-chlorophenylthio)-2′-*O*-Me-cAMP-AM (8-CPT-AM), which was purchased from Biolog Life Science Institute (Bremen, Germany).

### Myocyte preparation

Primary cultures of neonatal rat cardiac myocytes and adult rat ventricular myocytes were prepared as described in our previous publications [24, 25].

### Immunoblotting

Immunoblotting was conducted using commercially available antibodies. Signal transducer and activator of transcription 1 (STAT1), STAT3, phosphor-STAT1 (Tyr701) and phosphor-STAT3 (Tyr705) antibodies were purchased from Cell Signaling Technology (Danvers, MA). iNOS and SOCS3 antibodies were purchased from Immuno-Biological Laboratories Inc. (Minneapolis, MN) and Cayman Chemical (Ann Arbor, MI), respectively. Protein expression was quantified by densitometry.

### Real-time quantitative PCR

Total RNA was extracted using TRIzol Reagent (Invitrogen, Thermo Fisher Scientific, Carlsbad, CA ) according to the manufacturer’s protocol. Total RNA was reverse-transcribed with the SuperScript First-Strand Synthesis System for RT-PCR kit (Invitrogen) according to the manufacturer’s instructions.

mRNA expression of SOCS3 and SOCS1 was quantified by quantitative real-time PCR using the ABI-PRISM^®^ 7700 sequence detection system (Applied Biosystems, Thermo Fisher Scientific, Foster City, CA) with the SYBR Green PCR Master Mix. The primer pairs for SOCS3 and SOC1 were: SOCS3 (forward, 5′-CTGGACCCATTCGGGAGTTC-3′; reverse, 5′-AACTGGGAGCTACCGACCATTG-3′) and SOCS1 (forward, 5′-CTGCGGCTTCTATTGGGGAC-3′; reverse, 5′-AAAAGGCAGTCGAAGGTCTCG-3′). The relative amount of mRNA of SOCS3 and SOCS1 was normalized to 18S rRNA.

### Measurements of intracellular Ca^2+^ concentration and myocyte shortening

Freshly isolated adult rat myocytes were placed on a laminin-coated 3-cm dish and incubated with or without 8-CPT-AM (10 μmol/L) for 5 h in medium at 37 °C before dye loading or measurement of myocyte shortening.

Myocytes were loaded together with the fluorescent indicator fura-2 acetoxymethyl ester (fura-2 AM; Molecular Probes, Thermo Fisher Scientific, Eugene, OR) as described previously, with some modifications [26]. In brief, 5 μL of a 1 mmol/M stock solution of fura-2 AM (dissolved in dimethyl sulfoxide) was added to cells in 2.0 ml Tyrode solution (in mM: HEPES, 5; NaCl, 140; KCl, 5; MgCl_2_, 1; glucose, 10; CaCl_2_, 1.8; pH adjusted to 7.4 with NaOH) to give a final fura-2 concentration of 5 μmol/L. Cells were loaded for 20 min at room temperature, then washed in Tyrode solution twice to remove unincorporated fura-2 AM and incubated with or without the addition of IL-6 (30 ng/ml) for 30 min at room temperature before the response of intracellular Ca^2+^ to isoproterenol (ISO) was measured.

Conversion of fura-2 fluorescence ratio to intracellular Ca^2+^ concentration was performed as described previously, with some modifications [27, 28]. In brief, the fura-2-loaded cells were placed on the stage of an inverted microscope (Nikon Eclipse TE2000; Nikon Corp., Tokyo, Japan). To measure intracellular Ca^2+^ concentration, we alternatively illuminated myocytes with 340- and 380-nm light using a high-speed wavelength-switching device (Lambda SC; Shutter Instruments Co., Novato, CA), and images were recorded with an electron-multiplying charge-coupled device camera (Evolve 512; Photometrics, Tucson, AZ). The images were analyzed using image analysis software (NIS-Elements 3.1; Nikon Corp.). Regions of interest (ROIs) were selected in isolated adult rat myocytes, and fluorescence signals were monitored in the created ROIs in the presence or absence of 8-CPT-AM (10 μmol/L) for 30 s at baseline [29]. The ISO-induced elevation of the fluorescence signal was then monitored at increasing Ca^2+^ concentrations (10^−7^, 10^−6^ and 10^−5^ M) in the presence of ISO for 30 s. Mean fluorescence signals were converted to free Ca^2+^ concentration by accessing NIS-Element ratio dialogs.

Measurements of myocyte shortening in response to ISO were performed as described previously with or without the addition of IL-6 (30 ng/ml) for 30 min at room temperature before the measurements [30].

### Statistical analysis

All data were presented as mean ± standard error of the mean. Data were compared using Student’s *t* test when two samples were considered or using analysis of variance followed by Bonferroni posttest for three or more samples. Differences were considered to be significant at *p* < 0.05.

## Results

### Effects of various cytokines on the Jak-STAT pathway in cardiac myocytes

**Fig. 1:**
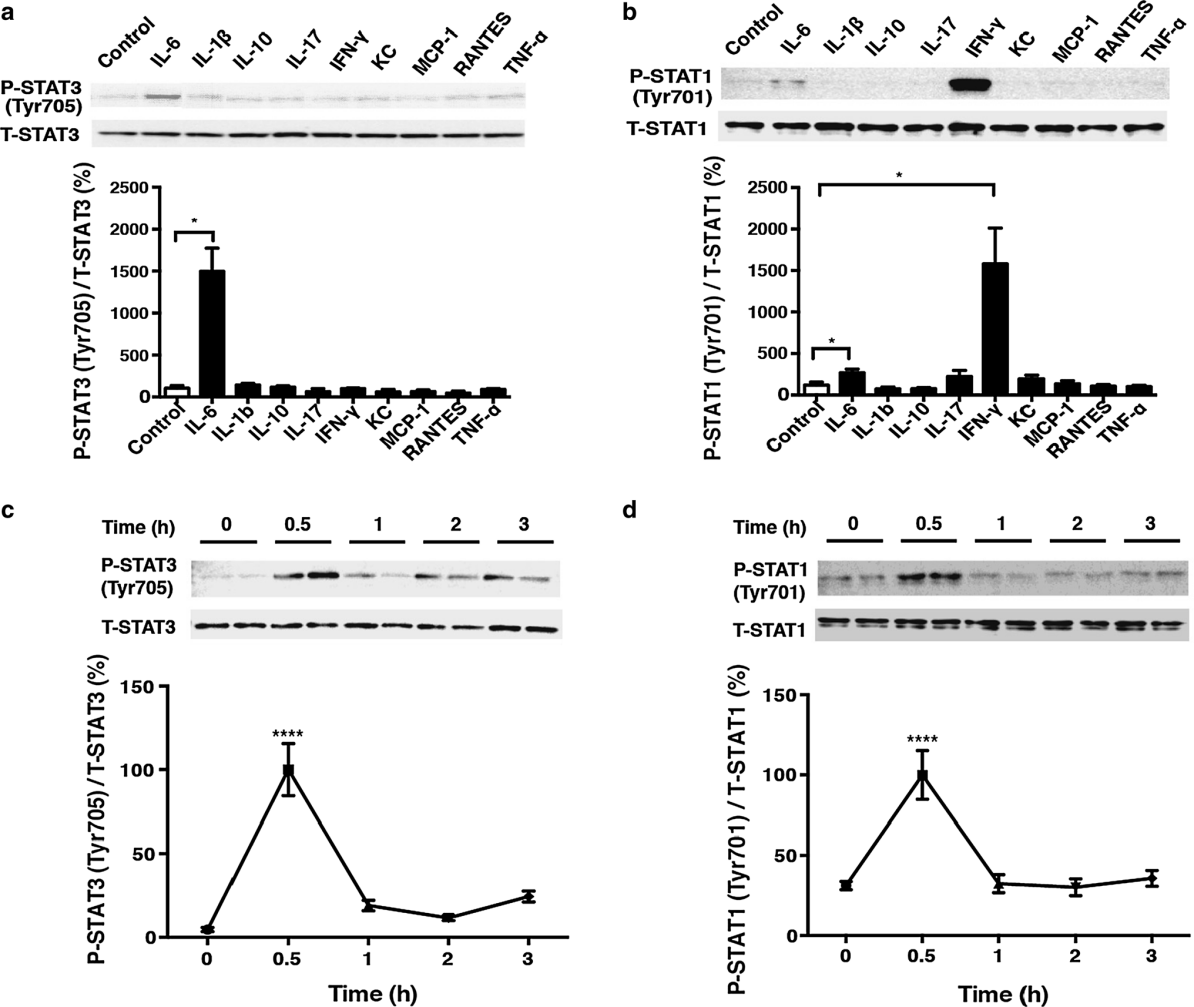
Effects of various cytokines on signal transducer and activator of transcription (STAT) phosphorylation and the time course of interleukin (*IL*)-6-mediated STAT phosphorylation. **a** Phosphorylation of signal transducer and activator of transcription 3 (*STAT3*) at Tyrosine 705 (*Tyr705*) was examined at 30 min after treatment with IL-6 (30 ng/ml), IL-1β (10 ng/ml), IL-10 (10 ng/ml), IL-17 (10 ng/ml), interferon gamma (*IFN-γ*; 10 ng/ml), keratinocyte-derived chemokine (*KC*; 50 ng/ml), monocyte chemotactic protein-1 (*MCP-1*; 10 ng/ml), RANTES (regulated upon activation, normal T cell expressed and secreted; 10 ng/ml), and tumor necrosis factor alpha (*TNF-α*; 10 ng/ml). The phosphorylation was upregulated by 14-fold in response to IL-6 (*n* = 4, *significantly different at *p* < 0.05, Student’s *t* test).* T-STAT* Total STAT,* P-STAT* phosphorylated STAT. **b** Phosphorylation of signal transducer and activator of transcription 1 (*STAT1*) at Tyrosine 701 (*Tyr701*) was examined at 30 min after treatment with IL-6 (30 ng/ml), IL-1β (10 ng/ml), IL-10 (10 ng/ml), IL-17 (10 ng/ml), IFN-γ (10 ng/ml), KC (50 ng/ml), MCP-1 (10 ng/ml), RANTES (10 ng/ml) and TNF-α (10 ng/ml). The phosphorylation was upregulated by 13-fold in response to IFN-γ and by 2-fold in response to IL-6 (*n* = 4, *significantly different at *p* < 0.05, Student’s *t* test). **c** Time course of IL-6 (30 ng/ml)-mediated STAT3 phosphorylation at Tyr705. Phosphorylation peaked at 30 min after treatment with IL-6 (30 ng/ml) and then decreased gradually [*n* = 4, ****significantly different at *p* < 0.0001 vs. 0 h, one-way analysis of variance (ANOVA)]. **d** Time course of IL-6 (30 ng/ml)-mediated phosphorylation of STAT1 at Tyr701. Phosphorylation peaked at 30 min after treatment with IL-6 and then decreased gradually (*n* = 4–6, ****significantly different at *p* < 0.0001 vs. 0 h, one-way ANOVA). Data are presented as the mean ± standard error of the mean (SEM)

 Lipopolysaccharide injection is known to increase the serum levels of cytokines, such as IL-1β, IL-6, IL-10, IL-17, interferon gamma (IFN-γ), KC (keratinocyte-derived chemokine), MCP-1 (monocyte chemotactic protein-1), RANTES (regulated upon activation, normal T cell expressed and secreted) and TNF-α, and thereby augments tyrosine phosphorylation of STAT3 in the liver and kidneys [31, 32]. However, the contribution of these cytokines to STAT phosphorylation in the heart has not been well studied. We first examined to what extent each cytokine contributed to the phosphorylation of STAT in neonatal rat cardiac myocytes. Phyosphorylation of STAT3 at Tyrosine 705 (Tyr705) was significantly and strikingly increased in response to IL-6 (14-fold) (Fig. 1a), but not to other cytokines, with phosphorylation peaking at 30 min after treatment with IL-6 (Fig. 1c). Phosphorylation of STAT1 at Tyrosine 701 (Tyr701) was significantly increased in response to IL-6 (2-fold) and IFN-γ (13-fold), but not to other cytokines **(**Fig. 1b), and IL-6-mediated phosphorylation of STAT1 also peaked at 30 min (Fig. 1d). Thus, among the various cytokines examined, our results show that IL-6 may play an important role in the activation of the Jak-STAT pathway, a major cytokine signaling pathway, in the heart.

### Effects of Epac on activation of the Jak-STAT pathway in response to IL-6

**Fig. 2:**
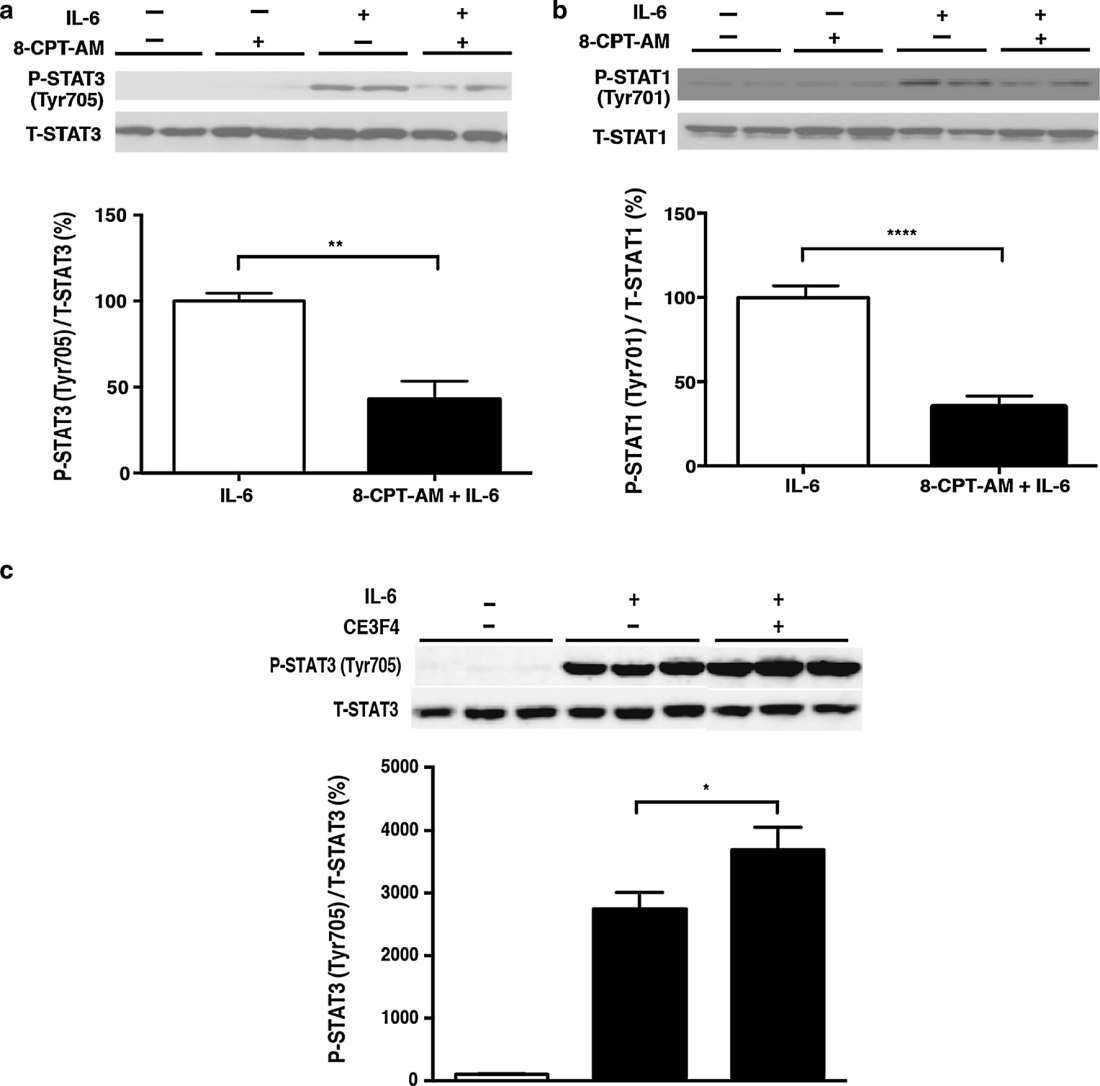
Effects of exchange protein activated by cAMP (Epac) on the activation of the Janus kinase–signal transducers and activators of transcription (Jak-STAT) pathway in response to IL-6. **a**, **b** Pretreatment of neonatal rat cardiac myocytes with 8-(4-chlorophenylthio)-2′-*O*-Me-cAMP-AM (*8-CPT-AM*; 10 μmol/L) for 5 h significantly reduced both IL-6-mediated phosphorylation of STAT3 at Tyr705 by 57 ± 11.5% (**a**) (*n* = 5–6, **significantly different at* p* < 0.01, Student’s *t* test) and IL-6-mediated phosphorylation of STAT1 at Tyr701 by 65 ± 6.8% (**b**) (*n* = 6–8, ****significantly different at *p* < 0.0001, Student’s *t* test). **c** Pretreatment of neonatal rat cardiac myocytes with Epac1 selective inhibitor CE3F4 for 30 min significantly enhanced IL-6-mediated phosphorylation of STAT3 at Tyr705 by 35 ± 13.0% (*n* = 6, *significantly different at *p* < 0.05, one-way ANOVA). Data are presented as the mean ± SEM

Epac1 has recently been shown to modulate cytokine signaling in non-cardiac cells, such as macrophages or endothelial cells, in vitro [22, 23, 33]. We thus examined the consequences of Epac activation on IL-6 signaling, as shown above, on cardiac myocytes. IL-6-stimulated phosphorylation of STAT3 and STAT1 was examined after treatment of the cells with 8-CPT-AM, an Epac-selective cAMP analog [34]. While 8-CPT-AM (10 μmol/L) had no effect on basal phosphorylation of STAT3 or STAT1 in neonatal rat cardiac myocytes, pretreatment with 8-CPT-AM for 5 h significantly diminished the IL-6-induced phosphorylation of STAT3 (Fig. 2a) and STAT1 (Fig. 2b). Thus, pharmacological activation of Epac did not alter basal phosphorylation, but it did potently inhibit IL-6-induced STAT phosphorylation. On the other hand, in the presence of Epac1 selective inhibitor CE3F4 (10 μmol/L), IL-6-induced STAT3 phosphorylation was significantly enhanced (Fig. 2c), suggesting that endogenous Epac1 inhibits IL-6-induced STAT3 phosphorylation.

### Epac activation increased SOCS3 and SOCS1 levels

**Fig. 3:**
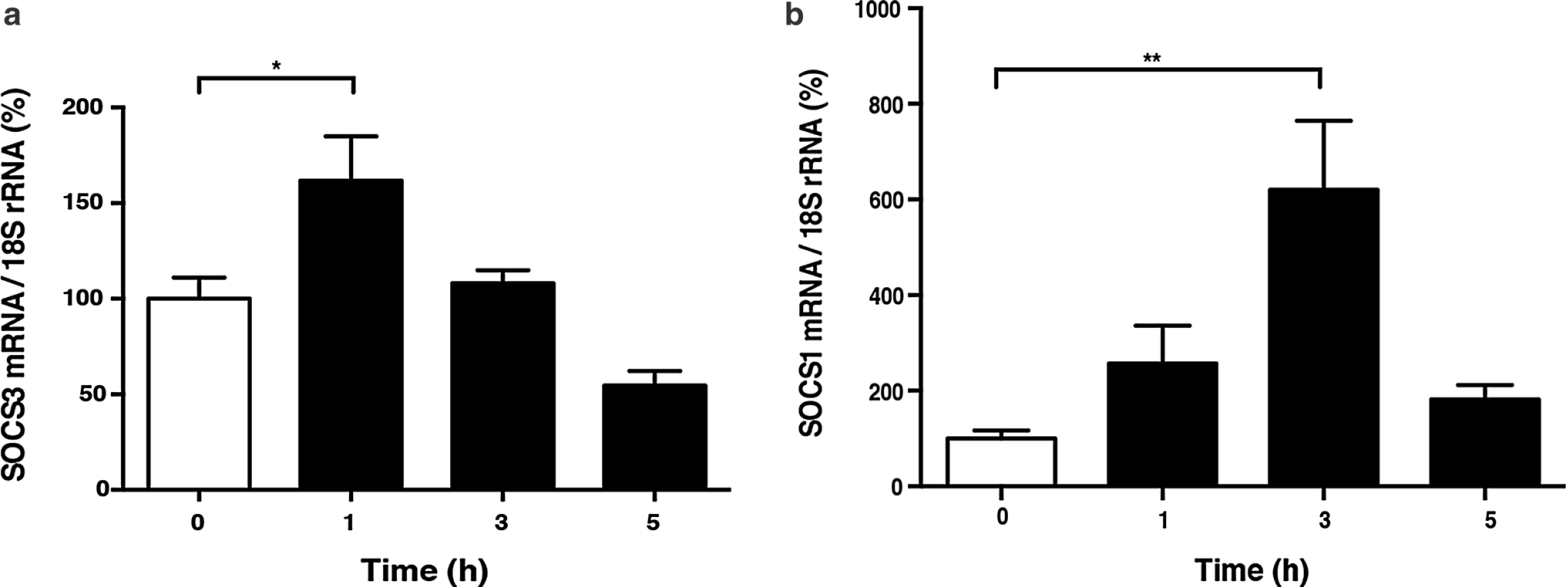
Time course of Epac-mediated expression of suppressor of cytokine signaling-3 and -1 (*SOCS3*,* SOCS1*, respectively) in cardiac myocytes. **a**, **b** mRNA expression of SOCS3 (**a**) and SOCS1 (**b**) was examined by quantitative real-time PCR in neonatal rat cardiac myocytes in the presence of 8-CPT-AM (10 μmol/L) for the indicated time. mRNA expression of SOCS3 and SOCS1 gradually increased with 8-CPT-AM treatment.** a** SOCS3 mRNA expression peaked at a significant increase of 1.7-fold at 1 h after 8-CPT-AM treatment (*n* = 4, *significantly different at *p* < 0.05 vs. baseline, one-way ANOVA). **b** SOCS1 mRNA expression peaked at a significant increase of 6.2-fold at 3 h after 8-CPT-AM treatment (*n* = 3–4, **significantly different at *p* < 0.01 vs. baseline, one-way ANOVA).* NS* Not significant. Data are presented as the mean ± SEM

Among the negative regulators of the Jak-STAT pathway, we found that the mRNA expression of SOCS3 and SOCS1 was increased by Epac activation (Fig. 3). SOCS3 mRNA expression was significantly increased at 1 h after 8-CPT-AM treatment (10 μmol/L) by 1.7-fold (*p* < 0.05, *n* = 4) (Fig. 3a), and SOCS1 mRNA expression was significantly increased at 3 h after 8-CPT-AM treatment (Fig. 3b) by 6.2-fold (*p* < 0.01, *n* = 3–4). These data suggest that Epac activation induces mRNA expression of SOCS3 and SOCS1 and that these molecules may function as negative regulators of the IL-6-mediated Jak-STAT pathway activation in the 8-CPT-AM-treated cardiac myocytes.

### Epac-mediated SOCS3 expression via phospholipase C/protein kinase C

**Fig. 4:**
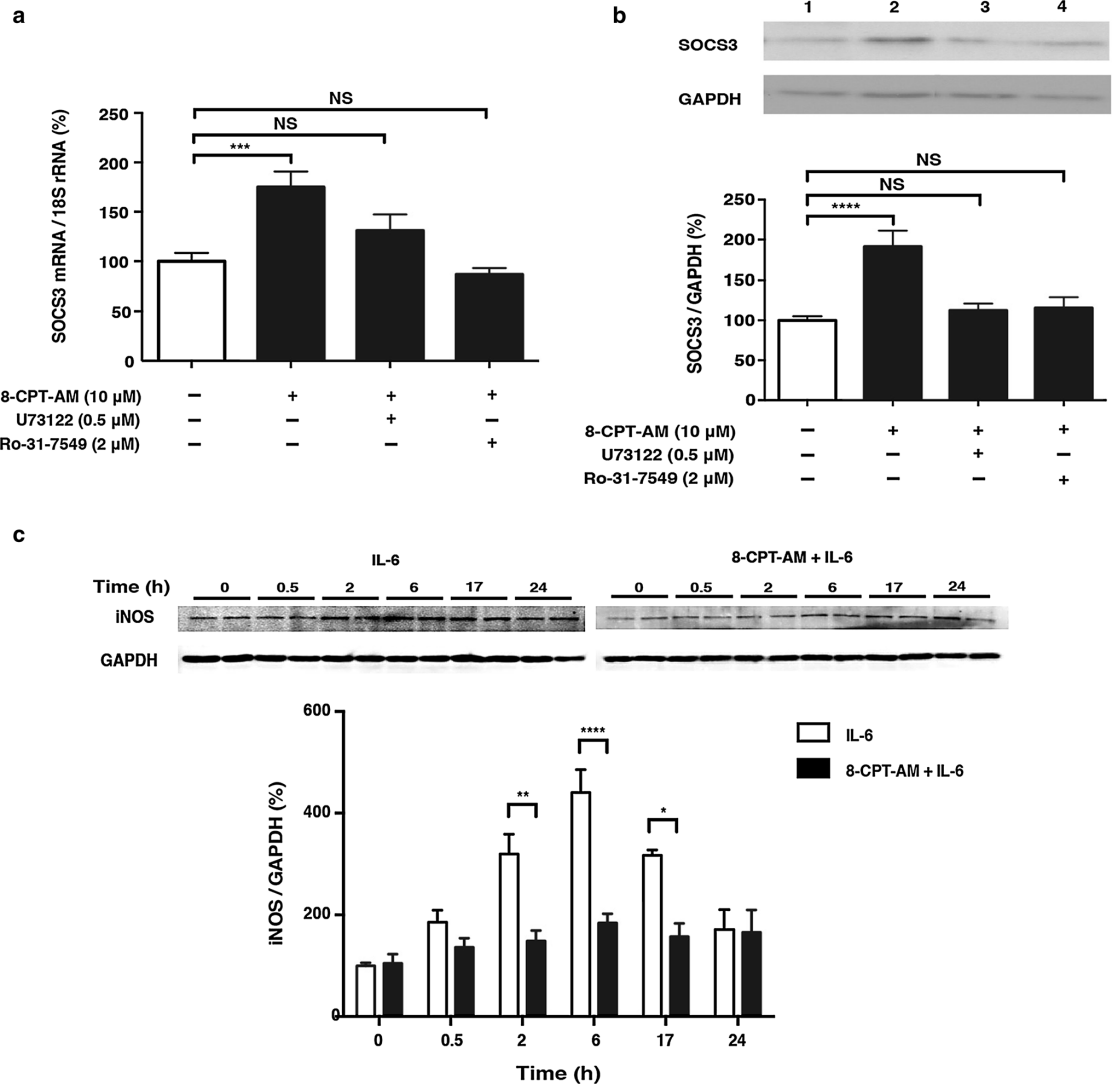
Effects of Epac activation on SOCS3 expression and IL-6 induced inducible nitric oxide synthase (*iNOS*) expression in cardiac myocytes. **a** SOCS3 mRNA expression, examined by quantitative real-time PCR in neonatal rat cardiac myocytes in the presence of 8-CPT-AM (10 μmol/L) for 1 h, was significantly increased by 1.8-fold. However, Epac-mediated SOCS3 upregulation was blunted by the addition of a phospholipase C (PLC) inhibitor (*U73122*, 0.5 μmol/L) or protein kinase C (PKC) inhibitor (*Ro-31-7549*, 2 μmol/L) (*n* = 6–10, ***significantly different at *p* < 0.001 vs. baseline, one-way ANOVA). **b** SOCS3 protein expression, examined by western blotting in the presence of 8-CPT-AM (10 μmol/L) for 5 h, was significantly increased by 2.2-fold in the presence of 8-CPT-AM. This upregulation was also blunted by the addition of PLC inhibitor or PKC inhibitor (*n* = 6–11, ****significantly different at *p* < 0.0001 vs. baseline, one-way ANOVA)**. c** Western blot analysis of iNOS expression in neonatal rat cardiac myocytes treated for the indicated times with IL-6 (30 ng/ml). iNOS expression increased gradually, peaking at 6 h. However, pretreatment with 8-CPT-AM (10 μmol/L) for 5 h inhibited the IL-6-mediated increase of iNOS protein expression (*n* = 4–7, *significantly different at *p* < 0.05, two-way ANOVA).* GAPDH* Glyceraldehyde 3-phosphate dehydrogenase. Data are presented as the mean ± SEM

We next examined the role of phospholipase C (PLC) and protein kinase C (PKC) in the increase of SOCS3 due to 8-CPT-AM treatment because a recent study indicates that those proteins are involved in cAMP elevation-induced SOCS3 accumulation in COS cells [35]. As expected, these increases were abolished in the presence of the PLC inhibitor U73122 (Fig. 4a) or PKC inhibitor Ro-31-7549 (Fig. 4b), suggesting that Epac activation increased SOCS3 expression via PLC/PKC mediation. Taken together, these results led us to hypothesize that Epac increases SOCS3- and inhibits IL-6-mediated phosphorylation of STAT in cardiac myocytes.

### Effects of Epac activation on IL-6-induced iNOS expression in cardiac myocytes

To address the functional consequence of Epac-mediated regulation of IL-6 signaling, we examined changes in molecules whose expression may be directly regulated by IL-6/Jak/STAT and which also play an important role in regulating cardiac function. We found that iNOS protein expression was increased by IL-6 (30 ng/ml) in a time-dependent manner, with a maximal increase of 4.5-fold at 6 h after IL-6 treatment (Fig. 4c), but that this increase was significantly inhibited by a 5-h pretreatment with 8-CPT-AM (10 μmol/L). These results led us to conclude that pharmacological activation of Epac inhibited the expression of iNOS.

### Effects of Epac on Ca^2+^ regulation and cell shortening in cardiac myocytes

Cardiac contraction and relaxation are influenced by the intracellular increase in Ca^2+^ during systole and its decrease during diastole. Ca^2+^ uptake by the sarcoplasmic reticulum is regulated via phospholamban phosphorylation and its release is regulated via the ryanodine receptor. We previously demonstrated that the basal and peak intracellular Ca^2+^ concentration is significantly decreased in isolated cardiac myocytes of Epac1-null mice compared with wild-type controls, with reduced phospholamban phosphorylation at the serine 16 residue [7]. In that same study, we also demonstrated that silencing Epac1 in cardiac myocytes attenuated ISO-mediated increases in phosphorylation of the ryanodine receptor on the serine 2808 and serine 2814 residues [7]. These data suggest that Epac1 activation, on its own, can cause changes in Ca^2+^ signaling and contractility in isolated cardiac myocytes.

**Fig. 5:**
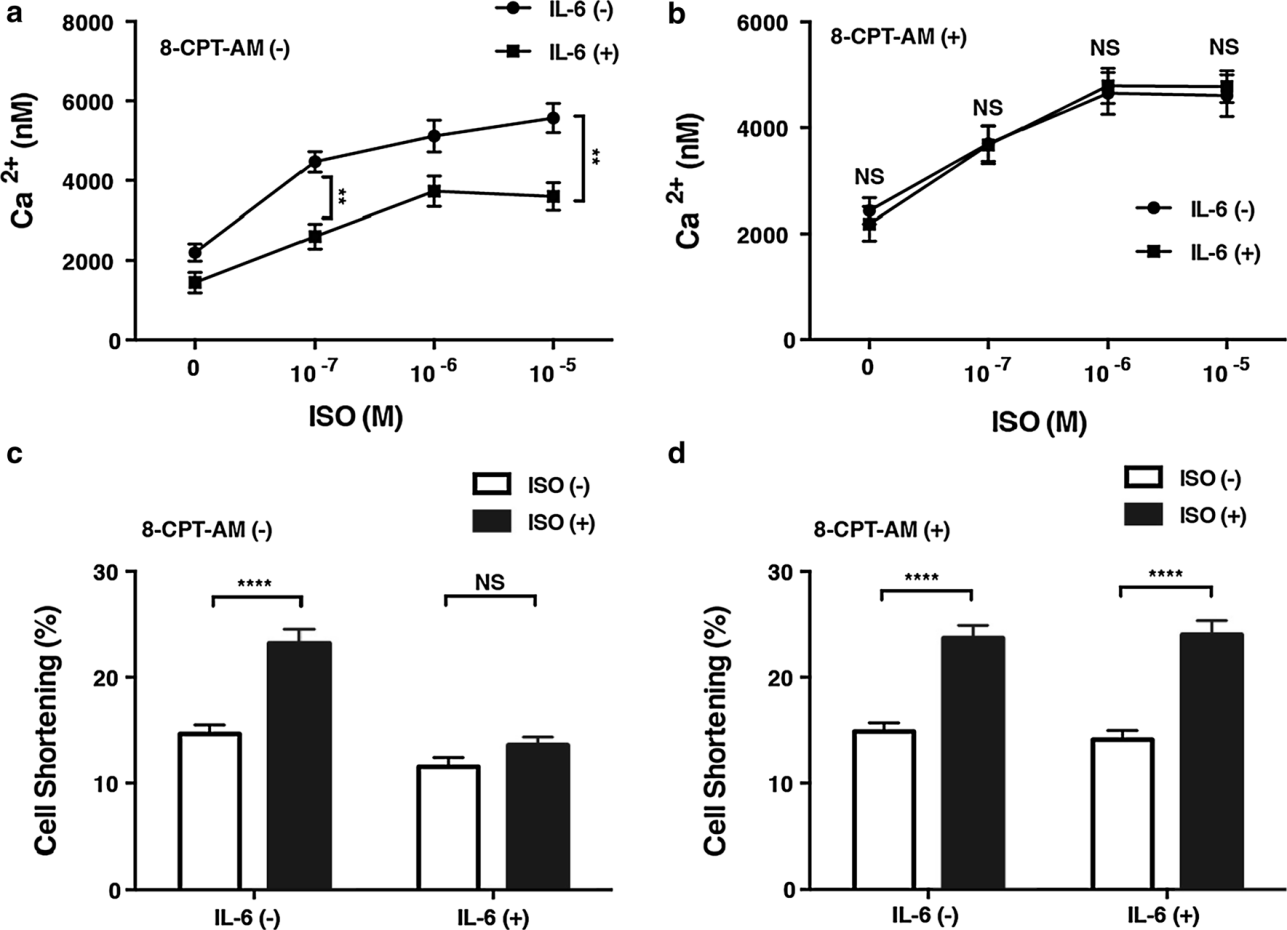
Effects of Epac activation on Ca^2+^ concentration (**a**,** b**) and contractility of cardiac myocytes (**c**,** d**). **a** Intracellular Ca^2+^ concentration in response to increasing concentrations of isoproterenol (*ISO*; 0–10^−5^ mol/L) in adult rat cardiac myocytes with or without a 6-h IL-6 (30 ng/ml) pretreatment. **b** The effect of a 5-h 8-CPT-AM (10 μmol/L) pretreatment on the ISO-induced increase of Ca^2+^. Pretreatment with 8-CPT-AM restored the increase of Ca^2+^ transients suppressed by IL-6 (*n* = 29–64, **significantly different at *p* < 0.01, two-way ANOVA). **c** Cardiac cell shortening at baseline and in response to ISO (10^−5^ mol/L).** d** Pretreatment with 8-CPT-AM (10 μM) restored cell shortening suppressed by IL-6 (30 ng/ml) (*n* = 24–31, ****significantly different at *p* < 0.0001, two-way ANOVA). Data are presented as the mean ± SEM

IL-6 has been shown to decrease the intracellular Ca^2+^ concentration and cell contraction in cultured chick cardiac myocytes through the induction of iNOS [36]. Our findings suggest that Epac can increase SOCS3 and SOCS1 expression, leading to inhibition of STAT3 phosphorylation and, consequently, iNOS expression. Consequently, it is possible that Epac antagonizes IL-6 with respect to regulation of the Ca^2+^ concentration and thus cell contraction. In our study, ISO increased the intracellular Ca^2+^ concentration in a dose-dependent manner in adult rat cardiac myocytes. This increase was significantly attenuated when the cells were pretreated with IL-6 (30 ng/ml) for 6 h, as expected (Fig. 5a). However, pretreatment with 8-CPT-AM (10 μmol/L) for 5 h abolished this attenuation by IL-6 (Fig. 5b). When myocyte shortening was examined as an index of cardiac contractility, ISO (10^−5^ mol/L) significantly increased cell shortening, and this increase was significantly attenuated by IL-6 (30 ng/ml) pretreatment for 6 h (Fig. 5c). Importantly, pretreatment with 8-CPT-AM for 5 h abolished this attenuation with IL-6 (Fig. 5d). Taken together, these findings suggest that pharmacological activation of Epac plays a key role in antagonizing IL-6, resulting in preservation of the Ca^2+^ regulation and thus preservation of cardiac contractility, at least in cultured myocytes.

## Discussion

Interleukin-6 inhibited the increase in intracellular Ca^2+^ concentration and thereby also myocyte shortening stimulated by ISO. This effect of IL-6 was diminished by pharmacological activation of Epac in cardiac myocytes, most likely through SOCS3/STAT/iNOS signaling. It has long been believed that the major target of catecholamine/cAMP signaling is PKA and that the PKA-mediated increase in myocyte contractility is the only mechanism compensating for cardiac dysfunction in heart failure. However, our findings suggest that there is another mechanism that involves Epac and that this mechanism may be able to restore cardiac myocyte function, at least in the context of cytokine-induced dysfunction. Thus, Epac appears to play an important role, unlike PKA, in the crosstalk between the sympathetic nervous system and pro-inflammatory cytokine signaling in the development of heart failure.

Of the various cytokines examined in our study, we found that IL-6 was the most prominent activator of STAT3 in cardiac myocytes and that IL-6-mediated STAT3 activation reduced the ISO-induced increase in Ca^2+^ concentration and cardiac contractility through the induction of iNOS. We also demonstrated that IL-6 significantly increased the phosphorylation of STAT1 even though IFN-γ is the most prominent activator of STAT1 in cardiac myocytes. Also, IL-6-mediated STAT1 activation was significantly decreased by the activation of Epac in cardiac myocytes. Previous studies have shown that STAT1 phosphorylation may be able to induce iNOS expression with the activation of p44/42 mitogen-activated protein kinase in cardiac myocytes [37]. Taken together, these results suggest that cytokine-mediated cardiac dysfunction is most likely mediated by nitric oxide production [14, 38]. We also found that this deterioration can be ameliorated by Epac activation. Cytokine levels are increased in patients with chronic congestive heart failure [14], in whom sympathetic nerve activity is also increased, suggesting that Epac activation may be beneficial in such patients, in addition to those with septic heart failure.

Our observation of an increase in SOCS3 mRNA/protein levels in cardiac myocytes in response to Epac activation is consistent with previous findings. For example, elevated cAMP levels have been found to promote SOCS3 accumulation in cultured cell lines, such as mouse embryonic fibroblasts or COS cells [22, 35, 39], most likely through the PLC/PKC pathway, as demonstrated in COS cells [35]. We also confirmed the increase in SOCS1 mRNA level in cardiac myocytes in response to Epac activation, which is known to exert negative feedback effects on cytokine signaling via the Jak-STAT pathway [40, 41]. More importantly, SOCS1-deficient mice are sensitive to LPS-induced lethal effects than wild-type control mice [42]. In our study, however, we demonstrated the functional significance of such changes, i.e., that Epac increased mRNA expression of SOCS3 and SOCS1 in cultured cardiac myocytes, thereby inhibiting IL-6/STAT/iNOS signaling and protecting the cardiac myocytes against cytokine-induced dysfunction.

Our findings suggest that the beneficial effect of the sympathetic nervous system involves not only enhancement of cardiac contractility through the classic cAMP/PKA pathway, but also inhibition of the Jak/STAT pathway via the newly described cAMP/Epac pathway. Epac signaling can attenuate pro-inflammatory cytokine-induced Jak/STAT signaling via SOCS3 and SOCS1, leading to the downregulation of iNOS, which is associated with cardiac dysfunction. It is known that cardiac dysfunction in sepsis is severe but that it may be restored almost completely in many cases [18, 19]. In cardiac myocytes, IL-6 treatment increases phosphorylation of STAT3 at the Tyr705 through the enhanced de novo synthesis of iNOS protein, leading to increased NO production and decreased cardiac contractility [38]. Furthermore, both IL-6-mediated STAT3 phosphorylation at Tyr705 and iNOS expression are blocked by AG490, a Jak2 inhibitor, or by genistein, a protein tyrosine kinase inhibitor, in cardiac myocytes [38]. IL-6 treatment also inhibits the ISO-induced increase in Ca^2+^ level and decreases cell contraction in cardiac myocytes [36]. However, pretreatment with *N* ^G^-monomethyl-l-arginine, an analog of l-arginine and a potent inhibitor of NO production, inhibits the IL-6-mediated decrease in intracellular Ca^2+^ transient and cell contraction in cardiac myocytes [36]. These previous data, together with the data from our study, indicate the causal relationship and functional significance of such changes, i.e., that Epac increased the expression of SOCS3 and SOCS1 in our cultured cardiac myocytes, thereby inhibiting IL-6/STAT/iNOS signaling and protecting the cardiac myocytes against cytokine-induced dysfunction.

Epac activation has been reported to increase myofilament Ca^2+^ sensitivity of isometric force through the increased phosphorylation of two key sarcomeric proteins, namely, cardiac myosin binding protein-C and cardiac troponin I [43], and it might also contribute to the Epac-mediated compensation of cardiac dysfunction in sepsis. We speculate that cAMP/Epac-mediated cardiac protection, as opposed to the cardiac damage caused by cytokines/endotoxin, may explain this beneficial effect, at least in part. Thus, we consider that the sympathetic nervous system can regulate cardiac function through multiple mechanisms.

In summary, we have demonstrated that Epac-mediated SOCS3 and SOCS1 expression protects the heart against cytokine-mediated cardiac dysfunction by inhibiting STAT3 phosphorylation and subsequent iNOS production. Our findings also suggest that Epac1 and its downstream pathway, including Jak2 or STAT3, may be an attractive target for treatment of cardiac failure in sepsis, and possibly cardiac failure in general, since cytokine-induced cardiac dysfunction plays an important role in the pathogenesis of cardiac failure [21].
